# Whole Genome Re-Sequencing Identifies a Quantitative Trait Locus Repressing Carbon Reserve Accumulation during Optimal Growth in *Chlamydomonas reinhardtii*

**DOI:** 10.1038/srep25209

**Published:** 2016-05-04

**Authors:** Hugh Douglas Goold, Hoa Mai Nguyen, Fantao Kong, Audrey Beyly-Adriano, Bertrand Légeret, Emmanuelle Billon, Stéphan Cuiné, Fred Beisson, Gilles Peltier, Yonghua Li-Beisson

**Affiliations:** 1 CEA, BIAM, Lab Bioenerget Biotechnol Bacteries & Microalgues, Saint-Paul-lez-Durance, 13108, France; 2 CNRS, UMR 7265 Biol Veget & Microbiol Environ, Saint-Paul-lez-Durance, 13108, France; 3 Aix Marseille Université, BVME UMR7265, Marseille, 13284, France; 4 Faculty of Agriculture and the Environment, University of Sydney, Australia

## Abstract

Microalgae have emerged as a promising source for biofuel production. Massive oil and starch accumulation in microalgae is possible, but occurs mostly when biomass growth is impaired. The molecular networks underlying the negative correlation between growth and reserve formation are not known. Thus isolation of strains capable of accumulating carbon reserves during optimal growth would be highly desirable. To this end, we screened an insertional mutant library of *Chlamydomonas reinhardtii* for alterations in oil content. A mutant accumulating five times more oil and twice more starch than wild-type during optimal growth was isolated and named *c*onstitutive *o*il *a*ccumulator 1 (*coa1*). Growth in photobioreactors under highly controlled conditions revealed that the increase in oil and starch content in *coa1* was dependent on light intensity. Genetic analysis and DNA hybridization pointed to a single insertional event responsible for the phenotype. Whole genome re-sequencing identified in *coa1* a >200 kb deletion on chromosome 14 containing 41 genes. This study demonstrates that, 1), the generation of algal strains accumulating higher reserve amount without compromising biomass accumulation is feasible; 2), light is an important parameter in phenotypic analysis; and 3), a chromosomal region (Quantitative Trait Locus) acts as suppressor of carbon reserve accumulation during optimal growth.

With increasing world population and diminishing fossil fuels, alternative fuel sources that are renewable and do not compete with food production for fresh water and arable land are highly sought after^1^,^2^. Due to their high growth rate and their intrinsic capacity (at least under certain growth conditions) to store large amounts of carbon rich compounds (i.e. starch and oils), microalgae have been investigated intensively as sources for biofuel production (bioethanol from starch; biodiesel from lipids)^1^,^2^,^3^. However, most algal species exhibit a maximal accumulation of starch and oil (i.e. triacylglycerols, TAGs) only when exposed to stress conditions, such as a nitrogen starvation, salinity, or high temperatures^4^,^5^,^6^,^7^,^8^,^9^.

The transition from optimal growth to carbon reserve accumulation requires changes at several systems levels including transcriptome, proteome and metabolome, highlighting the tight control that a cell exerts on the repartition of cellular carbon and energy usage^8^,^10^,^11^,^12^,^13^,^14^,^15^,^16^. Various genetic approaches have been applied to isolate factors involved in this transition. These reported regulatory proteins of lipid metabolism included a SQUAMOSA promoter-binding protein domain transcription factor (the nitrogen-responsive regulator 1, NRR1)^13^, a CXC domain-containing protein (the compromised hydrolysis of triacylglycerols 7, CHT7)^17^, and two members of the dual-specificity tyrosine phosphorylation-regulated kinase family, i.e. the triacylglycerol accumulation regulator1 (TAR1)^18^ and the starch degradation 1 (STD1)^19^. CHT7 is involved in the regulation of oil remobilization, however all three other proteins have been found to control oil amount under nitrogen starvation conditions. These three mutants (*nrr1*, *tar1*, and *std1*) provided regulatory clues as to how cells control oil accumulation in response to nitrogen starvation. Thus, these studies have mostly focused on the transitional changes from a healthy cell to a stressed cell. To our knowledge, no studies have been dedicated to investigate carbon reserve accumulation during optimal growth thus no regulatory gene or protein involved has been reported.

Under environmental conditions favorable for growth, genes of the lipid and starch biosynthetic pathways are expressed at low levels^10^, suggesting the occurrence of genetic program that suppresses carbon reserve formation. In this study, we applied a forward genetic screen in the green alga *Chlamydomonas reinhardtii* aiming at identifying new regulatory genes from the screening of mutants with altered cellular oil content during optimal growth. We used *C. reinhardtii* as a model because it has been proven as a suitable alga to study both starch and lipid metabolism^20^,^21^,^22^,^23^,^24^,^25^. We report here the detailed characterization of the mutant *coa1* standing for *c*onstitutive *o*il *a*ccumulator 1. This mutant accumulated increased oil and starch relative to the wild-type strain in a light-dependent manner. The isolation of this mutant should provide a molecular basis for our understanding of the control of TAG and starch accumulation during optimal growth.

## Results and Discussion

### Isolation of a Mutant with Higher Oil and Starch Content during Optimal Growth

**Figure 1:**
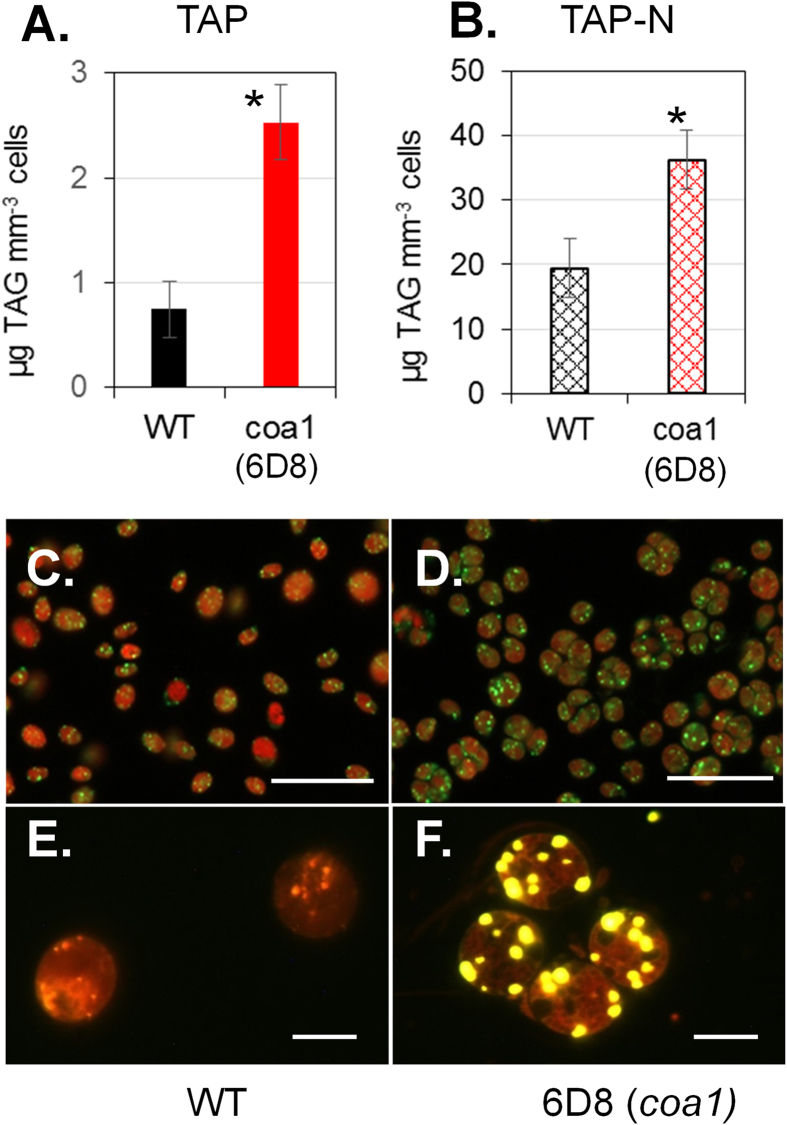
The *c*onstitutive *a*ccumulator 1 (*coa1*) mutant of *Chlamydomonas reinhardtii* over-accumulates oil during optimal growth in shake-flask cultures. (**A**) Triacylglycerol content as quantified by thin layer chromatography (TLC). Data are means of five biological replicates with 95% confidence intervals shown. Stars denote significant increase (Student’s t-test; *p* < 0.05). (**B**) Oil content in WT and *coa1* (6D8) mutant strains after nitrogen depletion for 3 days. Data are means of three biological replicates and two technical replicates, with 95% confidence intervals shown. Stars denote significant increase (Student’s t-test; *p* < 0.05). (**C,D**) Images of Bodipy-stained cells of WT and the mutant *coa1*. Scale bars = 50 μm. (**E,F**) Magnification of lipid droplets stained by Nile red. Scale bars = 5 μm. Cells were cultivated in shake flasks in standard TAP medium at a light density of 100 μmol photons m^−2^ s^−1^. Because the *coa1* mutant forms cell clusters, biochemical quantification is made based on total cellular volume instead of cell numbers. It is worth noting that for non-aggregated cells of *Chlamydomonas*, 1 mm^−3^ cellular volumes are equivalent to ~5 million cells.

As part of our effort to dissect lipid metabolic pathways and identify novel factors involved in carbon reserve formation, an insertional mutant library has been generated as described previously^26^. The parental line used here is the wild-type CC124. This mutant library was then screened for alterations in oil content during optimal growth conditions, according to the screening method described previously^27^. Overall, after screening 7,000 independent transformants by Nile red coupled to Flow cytometry, a mutant (initially named 6D8) showing higher level of Nile red fluorescence than WT under normal growth condition was isolated. Higher Nile red fluorescence is generally used as a probe for increased cellular oil content, and indeed, TAG quantification confirmed that the mutant 6D8 made 5 times more oil than WT under nitrogen-replete conditions (Fig. 1A). In addition to an increase in oil content, an increased number of lipid droplet (LDs) was observed in the mutant cells, as revealed by staining with two independent lipophilic dyes i.e. Bodipy (Fig. 1C,D) or Nile red (Fig. 1E,F). Furthermore, the mutant 6D8 accumulated 1.5 times more oil than WT after being starved for nitrogen for 3 days (Fig. 1B). Based on these findings, the mutant 6D8 was re-named as *c*onstitutive *o*il *a*ccumulator 1 (*coa1*). No significant difference could be detected in the major membrane lipids (Supplemental Figure S1A,B). Additionally, we also observed that during the recovery phase following a period of nitrogen starvation, the mutant was also impaired in oil remobilization (Supplemental Figure S2). This result could imply that the observed net increase in oil content in the mutant during optimal growth could be, at least partly, due to a defect in oil breakdown. But this and other possibilities should be tested in the future with labelling studies to measure the rate of oil turnover during optimal growth.

**Figure 2:**
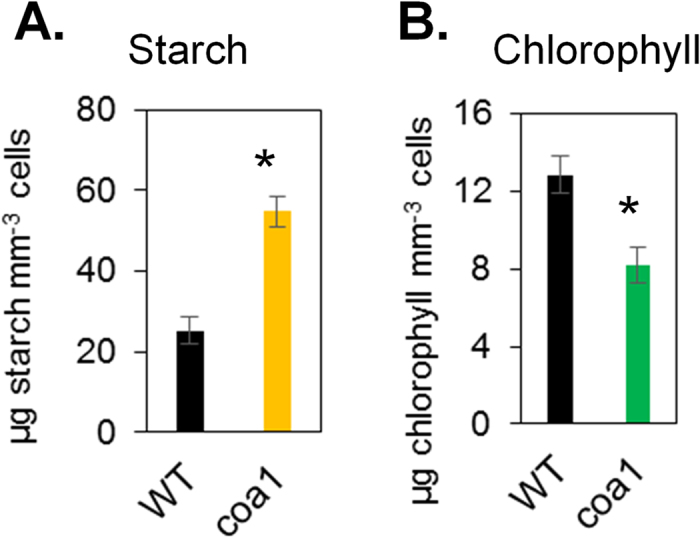
The *coa1* mutant accumulates twice more starch than WT during optimal growth in shake-flask cultures. (**A**) Starch content (**B**) Chlorophyll content. Data are means of six replicates with 95% confidence intervals shown. Stars denote significant increase (Student’s t-test; *p* < 0.05). Cells were cultivated in shake flasks in standard TAP medium at a light density of 100 μmol photons m^−2^ s^−1^.

Furthermore, the *coa1* mutant made almost twice more starch than its parental strain CC124 during normal growth (Fig. 2A), an increase from ~25 μg mm^−3^ to over ~50 μg mm^−3^. It is well known that the wild-type strains of *C. reinhardtii* store starch as a major form of polysaccharide reserve^20^, for reasons of simplicity, we have referred to polysaccharide reserves as starch throughout this paper. However it is worth noting here that the additional accumulation of polysaccharides observed in the mutant *coa1* could be starch or any other type of polysaccharide made of α-glucans, or a mixture of both. Under the same cultivation condition, *coa1* mutant contained less chlorophyll than WT (Fig. 2B). The parallel increase in intracellular oil and starch observed in the *coa1* mutant suggests that a common mechanism underlies regulation of these two storage compounds. This regulatory mechanism would be affected in the mutant, resulting in an increased carbon flux towards reserve formation.

### The *coa1* Mutant Forms Palmelloid Structures but Shows Similar Growth Rate as WT

**Figure 3:**
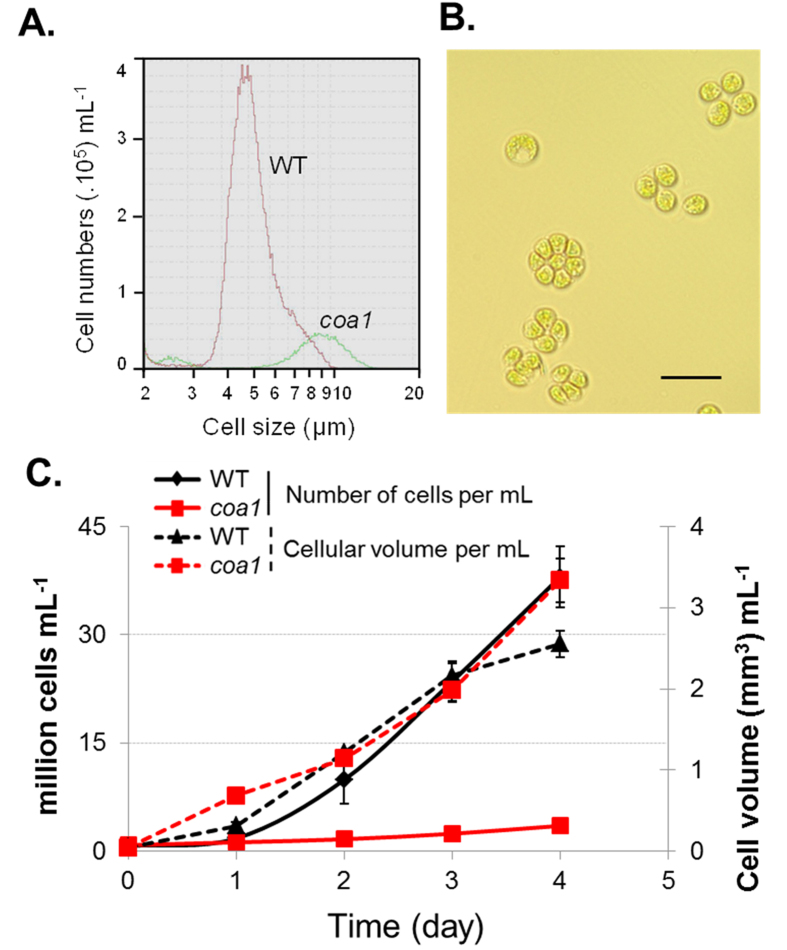
Growth characteristics of the *coa1* mutant and WT. (**A**) Population of WT and *coa1* mutant cells was monitored using a cell counter. The mutant cells appear much bigger (~9–10 μm) than WT cells (~5–6 μm) under the same cultivation conditions. (**B**) Clusters of the *coa1* mutant cells were observed under light microscope. Bars = 10 μm. (**C**) Cell growth for WT and *coa1* mutant cells were monitored based either on cell number (solid lines) or on cellular volume (dashed lines). Cells were grown in shake flasks in standard TAP medium at a light density of 100 μmol photons m^−2^ s^−1^. Error bars represent standard deviation based on three biological replicates. Abbreviations: TAP, Tris-Acetate Phosphate.

During cell counting, the *coa1* mutant was found to form aggregates, showing much bigger particles in diameter than WT (Fig. 3A). The aggregated clusters were also observed under a microscope (Fig. 3B). Such a phenotype has been previously described in *Chlamydomonas* mutant strains, and these are referred to as palmelloid colonies^28^. Formation of palmelloid colonies seem to be common^29^, and has been suggested as part of microalgal self-defense against stressors^30^. In an attempt to avoid cell aggregation, strategies were employed to assess whether this phenotype was due to growth conditions, or whether it was intrinsic to the mutant. Colonies were picked and re-plated successively in order to recover a single cellular organization, and also floating cells were recovered from the culture medium by allowing cells to settle at room temperature without shaking for an hour. These isolated cells were transferred to new cultures, thus selecting only those individual cells that are lighter and motile with active flagella, however the growth phenotype persisted. Due to the difficulty in accurately counting clustered cell aggregates, cellular volume has been used for cellular quantification rather than cell numbers. On a total cellular volume basis, similar growth rates were observed between the *coa1* mutant and WT (Fig. 3C).

### The High-Oil and High-Starch Phenotype of the *coa1* Mutant is Dependent on Light

**Figure 4:**
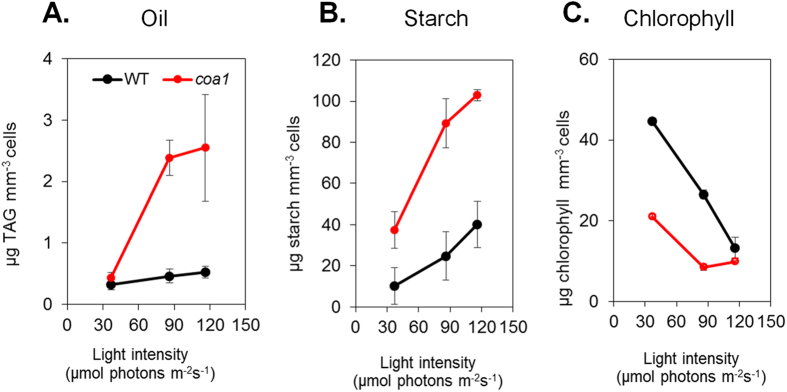
The *coa1* phenotype is positively linked to higher light intensity in photobioreactors operated as turbidostat. (**A**) TAG content in cells cultivated under increasing light irradiance. (**B**) Starch content in cells cultivated under increasing light irradiance. (**C**) Chlorophyll content in cells cultivated under increasing light irradiance. It is important to note that 40 μmol photons m^−2^ s^−1^ in the PBR set-up being approximately equivalent to 160 μmol photons m^−2^ s^−1^ in the flask batch set-up, roughly a 4-fold change. Data are means of three replicates, and error bars denote 95% confidence intervals. Cells were cultivated in PBRs under strict photoautotrophic conditions maintaining a constant OD_880 nm_ = 0.4 (eq. = 2 million cells mL^−1^ for WT).

When comparing oil contents of wild-type and mutant cells grown in flasks, we sometimes observed big variations in measurements despite the fact that cells were apparently cultivated under similar conditions. We then hypothesized that such variations may be related to a light effect, as the light absorbed by algal cells is greatly reduced during the time course of a batch culture performed in flasks, due to a shading effect as cell density gets higher^31^. In order to test this hypothesis, we cultivated the mutant and WT in well-controlled conditions of illumination, by using photobioreactors (PBRs) operated as turbidostats under photoautotrophic conditions. In this experimental set-up, cell density was assessed by measuring the turbidity (OD_880nm_) and was maintained at a constant value (OD_880nm_ = 0.4 corresponding to 2 million cells mL^−1^) by step-wise injection of fresh culture medium. Using this system, photoautotrophic cultures can be maintained at physiological steady states under different light regimes. It should be mentioned here that because PBRs are radially illuminated and cell density maintained at a relatively low level, the light required in this set-up to observe physiological effects of the same magnitude as in flasks are much lower (a factor of 4 is observed: i.e. 40 μmol photons m^−2^ s^−1^ in the PBR set-up being approximately equivalent to ~160 μmol photons m^−2^ s^−1^ in shake flask culture). Increasing light intensity from 40 to 120 μmol photons m^−2^ s^−1^ had very little effect on intracellular oil levels in the WT strain, but led to an increase in oil content from 0.5 to 2.5 μg mm^−3^ in mutant cells (Fig. 4A).

**Figure 5:**
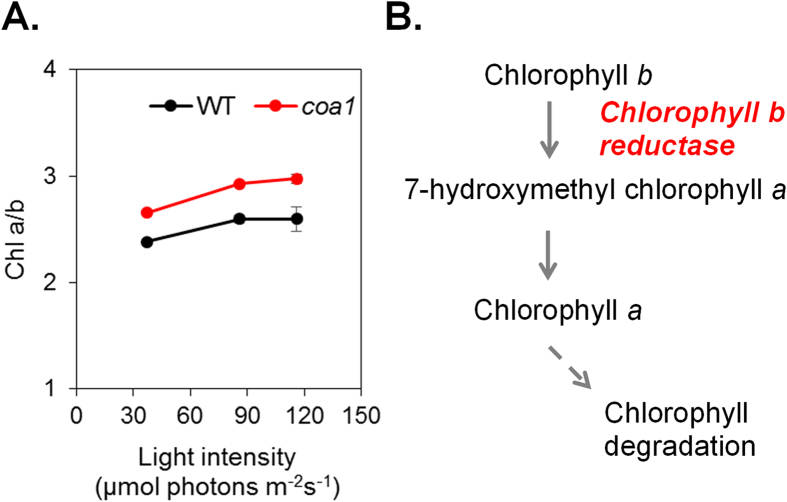
Alterations in the ratio of chlorophyll *a* versus chlorophyll *b* (Chl *a*/*b*). (**A**) Difference in Chl *a*/*b* ratio and its evolution in response to increasing light intensity. (**B**) The chlorophyll cycle and the requirement for a chlorophyll *b* reductase. Data are means of three biological replicates with two technical replicates each. Error bars represent standard deviations. Chl: chlorophyll.

Intracellular starch increased in both WT and the *coa1* mutant with increasing light intensity, but the increase was more pronounced in the mutant (Fig. 4B). Such an effect of light on starch accumulation has been previously reported for different species, including duckweed^32^, *Scenedesmus sp.*^33^ and *Chlorella sp.*^31^, and also in *Chlamydomonas reinhardtii*^34^, however a physiological explanation has not been put forward. When cells were grown under low light, the chlorophyll content was about 50% lower in the *coa1* mutant than in the WT (Fig. 4C). However, the efficiency of the photosynthetic machinery was only partially affected in *coa1*, as shown by photosystem II yields measured under various light intensities by means of chlorophyll fluorescence (Supplemental Figures S3). As generally observed during high light acclimation^35^, the chlorophyll content of both strains decreased (Fig. 4C), reaching a similar value under high light. It is well known that when algae or plants are grown under high light (HL) intensity, photosystems develop smaller antennae sizes and contain lower amounts of Chlorophyll *b* (Chl *b*) thus resulting in an increase in the chlorophyll *a*/*b* (Chl *a*/*b*) ratio^36^. An increase in the Chl *a*/*b* was observed in both strains under HL, the Chl *a*/*b* ratio being however higher in the *coa1* mutant than in the WT (Fig. 5A). It is important to note that when cells were grown in the dark both strains displayed similar Chl *a*/*b* ratios (Supplemental Figure S4) showing that this effect is related to light acclimation. Altogether, these data suggest that the *coa1* mutant is affected in its ability to regulate the antenna size and the chlorophyll content in response to HL conditions. This interpretation is supported by the immunoblot analysis which shows lower amounts of the light harvesting complex (LHCII) antenna protein LHCB5 in the *coa1* in comparison to the WT, with LHCB5 decreasing in response to HL acclimation in both strains (Supplemental Figure S5).

The increased starch and the decreased chlorophyll content observed in the mutant together suggest that the *coa1* mutant is somehow more sensitive to light as we have observed repeatedly, i.e. behaving under lower light similarly to WT under high light. Moreover, light is found to trigger higher oil accumulation in the mutant under increasing light intensity, whereas oil content increased only slightly in the WT under the light range tested. The *coa1* mutant therefore provides a means to probe the link between light intensity and reserve formation, a phenomenon which has sometimes been observed in the literature^37^ but has rarely been explored at a mechanistic level.

### The Phenotype of the *coa1* Mutant Results from a Single Insertional Event

**Figure 6:**
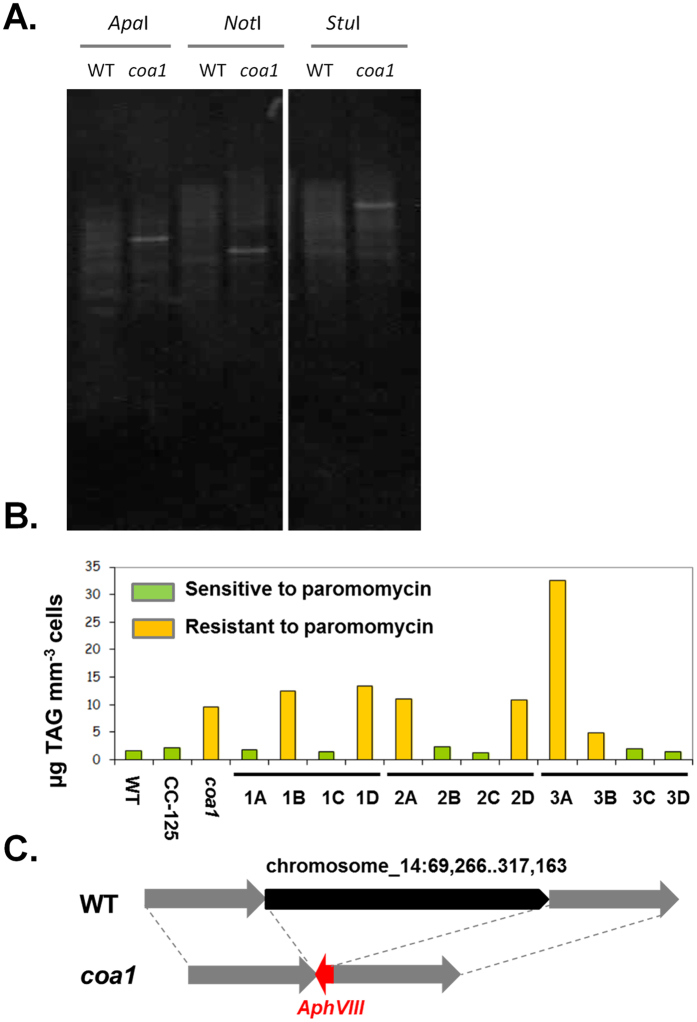
Genetic analyses confirm a single insertion in the *coa1* genome. (**A**) DNA blotting of digested genomic DNA of WT and *coa1* mutant with three different enzymes and then hybridized with the *AphVIII* probe. (**B**) Oil content for the progenies issued from tetrad analyses of *coa1* and CC124. (**C**) Whole genome re-sequencing identifies >200 kb deletion in chromosome 14 between coordinates 69,266 and 317,163. T: tetrad.

Insertional mutagenesis can lead to insertion of several copies of DNA cassettes randomly in a genome^38^,^39^. As a first step to determine whether the *coa1* phenotype is genetically linked to the insertion of the paromomycin cassette, we performed a DNA/DNA hybridization analysis (Southern blot). After digesting the *coa1* genomic DNA with several restriction enzymes independently, a single hybridizing band was observed in all cases using a probe specific to the DNA cassette harboring paromomycin gene *AphVIII*. This suggested that there is only one insertion of *AphVIII* cassette in the *coa1* genome (Fig. 6A).

As a second step, we performed genetic backcrosses between *coa1* (*mt*^−^) and the wild-type strain CC125 (*mt*^+^) which is the *mt*^−^ strain mostly closely related to the *coa1*’s progenitor (CC124). From the analysis of 3 complete tetrads (Fig. 6B) and 9 incomplete tetrads (Supplemental Figure S6A), we found that all paromomycin-resistant strains accumulated higher oil amount than paromomycin-sensitive strains and showed persistent palmelloid colony formation. Chlorophyll content in the three complete tetrads also showed the same segregation as cellular oil content, i.e. those progenies sensitive to paromomycin also contained less chlorophyll, which was already obvious from visual inspection of the color of the culture (Supplemental Figure S6B). No strain issued from this cross harbored the constitutive TAG accumulating phenotype in the absence of paromomycin resistance, thus indicating a genetic link between the oil phenotype and the single insertion of the antibiotic cassette.

### Whole Genome Re-Sequencing Identifies a Large Deletion in Chromosome 14

Although Southern blot analysis revealed a single insertional event occurred in the *coa1* mutant (Fig. 6A), two distant sites of insertion were identified on chromosome 14 by sequencing flanking regions of the insertion (one at 69,200 bp and the other at 315,218 bp) using Genome Walker technique. In order to understand how a single insertion event could lead to the identification of two distant sites, we re-sequenced the whole genome of *Chlamydomonas*. The genome sequencing generated 34257132 clean reads, with 90 bp per read and has a calculated coverage of 23.7x the whole *Chlamydomonas* genome (Table 1).

Genomic reads were paired using FASTQ joiner^40^ and mapped to the *C. reinhardtii* genome^41^ (version 4.3) using the BAM tools package available on the Galaxy project website (https://main.g2.bx.psu.edu/). Global and local coverage was determined by analysis of BAM output by GATK (Genome Analysis Tool Kit). The genomic region between 69,200 bp and 315,218 bp compared to the reference *Chlamydomonas* genome version 4.3 revealed low read coverage in the region of the insertion/deletion (mean coverage depth of 1.6, compared to a mean coverage depth of 25.4 for the entire chromosome 14). Peaks of high read coverage in the region between 69,200 bp and 317,163 bp on the reference genome were identified as highly repetitive motifs found elsewhere in the *Chlamydomonas* genome. Furthermore, reads mapped to the 3′ and 5′ end of the gene cassette, and reads paired to those which mapped to the cassette revealed that the genomic loci adjacent to the gene cassette had sequence identity to 69,200 bp and 315,218 bp on chromosome 14.

Taken together, the whole genome re-sequencing analysis and flanking genomic DNA sequencing by Genome Walker revealed that a single complete antisense *AphVIII* cassette was inserted in a 3′ –>5′ orientation between the 69,200 bp and 317,163 bp on chromosome 14, resulting in a substantial genomic deletion of >200 kb in chromosome 14 between the coordinates 69,266 and 317,163 (Fig. 6C). Chromosomal deletions caused by insertional mutagenesis in *C. reinhardtii* have previously been reported^17^,^35^. Within the >200 kb region, 41 putative protein-coding genes have been found (Table 2). The phenotype associated with *coa1* could thus be attributed to either the result of one of the genes removed in the deleted locus of the genome, or a combinatorial effect of multiple genes. This and other possibilities are discussed in the next section.

### Suppressors of Carbon Reserve Accumulation Within Chromosome_14:69,266.317,163

The link between the lesion on chromosome 14 and the observed increased level of TAG and starch under high light conditions suggest that carbon reserve formation is repressed in optimal growth in wild-type strains. In the absence of this chromosomic region (the *coa1* mutant), the cellular homeostasis of carbon reserves is disturbed, thus creating strains that over-accumulate oil and starch simultaneous to growth. Furthermore, it is found that one or more of the genes encoded on the same region may be key in the orchestration of the light response in *C. reinhardtii*. The reason *Chlamydomonas* exhibits an increased proportion of TAG and starch during high light growth remains poorly defined, and may remain difficult to analyze without the access to genome editing in *Chlamydomonas*, however the generation of an indexed genome-wide mutant library for *C. reinhardtii* (http://jonikaslab.dpb.carnegiescience.edu/chlamy-mutant-library)^42^ may aid in more rapidly elucidating which of these genes encode key functions in regulation of TAG homeostasis during high light.

While there is little information known about the proteins in the locus of interest (Table 2), some proteins do deserve particular note due to their potential involvement in the observed phenotype. For example, the presence of a lipase-like protein, a putative lipid transfer protein or a Coenzyme A binding protein in this region could perhaps contribute to the observed oil content phenotype in *coa1* mutant. The presence of a Myb domain-containing putative DNA binding protein is another example. Homologs of these proteins have been identified as potential key players in the carbon concentration mechanism (CCM) as transcription factors involved in the response to the physiological pressure of low carbon growth^43^. A gene encoding a chlorophyllide *b* reductase (Cre14.g608800) is also located in this chromosomic region. Chlorophyllide *b* reductase catalyzes the first step in the degradation of chlorophyll *b* in the chlorophyll cycle^44^. Its deletion in the *coa1* mutant may have perturbed the chlorophyll cycle thus explaining the altered chlorophyll content and chlorophyll *a*/*b* ratio observed in the mutant (Fig. 5B). Increase in chlorophyll *a*/*b* ratio is an integral feature of cells’ acclimation to high light conditions^45^. Although molecular mechanisms remain to be elucidated, the mutant *coa1* displays several phenotypic features (such as decreased chlorophyll content, increased chlorophyll *a*/*b* ratio and increased intracellular starch), indicating a higher sensitivity to light that may contribute to an increased overall flux in cellular carbon and an increase in oil content.

### Outlook

One of the major obstacles to economically feasible production of algal fuel is the requirement of nitrogen starvation or other stress conditions for higher reserve accumulation^2^. Here we isolated a mutant of *Chlamydomonas*, *coa1*, which accumulates five times more oil and twice more starch than the WT during optimal growth. To our knowledge, this is the first report of such a mutant accumulating higher oil amount in parallel to optimal growth for the green alga *C. reinhardtii*. Through the study of the mutant *coa1*, we also pointed out a missing link underlying the regulation of reserve accumulation under high light conditions. Molecular characterization of the *coa1* mutant identified a QTL responsible for the observed phenotype. Detailed examination of the genes, via cross-references to published transcriptomic datasets (Table 2)^34^, encoded in the missing region should help understanding the molecular mechanisms that led to increased amount of carbon reserves in the mutant, and should provide molecular tools for uncoupling lipid accumulation from impairment in cell growth. This report in conjunction with the availability of genome-wide indexed mutant libraries (http://jonikaslab.dpb.carnegiescience.edu/chlamy-mutant-library)^42^, and systems analysis of cells’ response to various stresses^10^,^13^,^34^, should make it faster to assign the *coa1* phenotype to particular gene(s) located within this region on chromosome 14. Thus the isolation of the *coa1* mutant demonstrates that mutants over-accumulating carbon reserves under non-stress conditions can be isolated and further provides a list of candidate genes involved in suppressing carbon reserve accumulation during optimal growth.

## Methods

### Strains, Growth Conditions and Generation of Insertional Mutants

*Chlamydomonas reinhardtii* wild-type strain CC124 (*mt*^−^ *nit1 nit2*), obtained from the Chlamydomonas resource center (http://chlamycollection.org), was used to generate the insertional mutant library in our laboratory as described previously in Nguyen *et al*.^26^. Briefly, the antibiotic resistance gene *AphVIII* was inserted into the genome of *C. reinhardtii* via electroporation^46^. Paromomycin-resistant clones were screened for alterations in oil content by Nile red staining coupled to Flow Cytometry as described in detail in Cagnon *et al*.^27^. Unless otherwise stated, all strains were cultivated at 25 °C in Tris-acetate-phosphate media (TAP) in shake flasks kept in incubators (Multitron, Infors HT, Switzerland) under continuous illumination (120 μmol photons m^−2^ s^−1^) with shaking (120 rpm)^28^. For nitrogen (N) starvation experiments, log-phase grown cells were centrifuged for 3 min at 1000 *g*, cell pellets were washed twice in TAP-N media, then resuspended into TAP-N for 3 days.

### Cell Counting and Microscopy

Cell numbers, sizes and total cellular volumes were quantified by the use of a Beckman Coulter Multisizer 4 (Multisizer^TM3^ Coulter Counter, Beckman Coulter, USA).

To observe lipid droplets, cells were first stained with a solution of Nile red (at a final concentration of 0.1 μg mL^−1^ in methanol; Sigma, Saint Louis, USA)^47^ or Bodipy (Bodipy 515, Invitrogen) at a final concentration of 1.5 μg mL^−1^ from a stock solution of 1 mg mL^−1^ in DMSO^48^. After incubation at room temperature for 20 min, cells were visualized under a Leica DMRXA epifluorescent microscope (Leica Microsystems, Germany). Flow cytometry measurement of Nile red fluorescence has previously been described in detail^27^.

### Cultivation in Photobioreactors (PBRs)

PBR cultures were cultivated in minimal medium (MM, i.e. without buffer neither carbon source)^28^ in automated ‘Biostat A Plus’ photobioreactor systems (Sartorius Stedim Biotech) equipped with a biomass probe (Excell probe, Exner; measuring OD_880 nm_ with a 2-cm light path) and operated as turbidostats. Cells were maintained at a constant density (OD_880nm_ = 0.4) throughout experimentation by automated injection of fresh media (Stepdos FEM03TT18RC; KNF) and ejection of excess biomass through a waste tube. Culture pH was maintained constant by addition of 0.2 N HCl or 0.2 N KOH using the Biostat module. A gas mixture containing air plus 1.8% CO_2_ was generated using two mass flow meters (EL flow; Bronkhorst) and bubbled into the PBRs at a flow rate of 0.5 L min^−1^. Eight fluorescent bulbs (Osram Dulux L 18 W) placed around the PBR were used to supply light in a range of light intensities up to 400 μmol photons m^−2^ s^−1^.

### Lipid Extractions and Lipid Quantifications by Thin Layer Chromatography (TLC)

A modified Bligh and Dyer method^49^ was used during this work to isolate total cellular lipids as described previously in Siaut *et al*.^4^. Briefly, samples were vortexed in quenching solution (1 mM EDTA, 0.15 M acetic acid) in a glass tube, then to which 3 mL of methanol:chloroform (2:1 v/v) was added. Samples were then vortexed for 10 min, 1 mL of chloroform and 0.8 mL of KCl (0.8% v/v) was added, and samples were vortexed briefly. Phases were separated by centrifugation at 600 *g* for 2 min at 4 °C in a bench top centrifuge. The lower phase was then isolated and 1 mL of hexane was added to re-extract the lipids, and samples were vortexed again for 10 min and phases were separated. The upper phase containing lipids was then transferred to the chloroform extract using a glass Pasteur pipette, and the rest was discarded. The combined chloroform and hexane extracts were then dried under a flow of nitrogen gas and re-suspended in an appropriate amount of solvent for subsequent analyses. Cellular content of lipid classes were quantified with Thin Layer Chromatography (TLC) using an automated “High-Performance Thin-Layer Chromatography” platform (CAMAG). These procedures were carried out as described previously^4^.

### Starch and Chlorophyll Quantification

A given number of cells (~2 million cells) were harvested by centrifugation (13000 *g* for 10 min) and cell pellets were resuspended in 1 mL methanol. The mixture was kept at −20 °C for at least 1 h before the organic phase was taken for chlorophyll quantification using spectrophotometer^50^. The pellet was air dried under a fume hood, and resuspended in MilliQ water, and autoclaved for 20 min at 121 °C. Amyloglucosidase (0.2 U, Roche) was added and samples were incubated for 1 h at 60 °C. Samples were briefly centrifuged, and measured for glucose concentration using a YSI2700 select sugar analyzer (YSI Life Sciences, Yellow Springs, USA)^4^.

### Mating and Tetrad Dissection

The *coa1* mutant was back-crossed with the wild-type CC125 (*mt*^+^). Genetic crosses were performed following the protocol described previously^51^. Briefly, *Chlamydomonas* strains of *mt*^*−*^ and *mt*^+^ were cultivated in TAP medium until mid-log phase. Cells were gently centrifuged at 360 *g* for 3 min, washed twice with TAP-N medium and resuspended in the same medium. Cultures were kept in TAP-N for 24 h at 25 °C under shaking. Cells with opposite mating types were mixed together in a glass tube (1 mL:1 mL) and then the tubes were kept in an incline position in a laminar hood for 3 hr. The mixture (1 mL) was then spread into center of TAP plates containing 3% agar (w/v), the plates were dried and kept under low light overnight. Then the plates were wrapped in aluminum foil, kept in the dark for 5–10 days to induce zygote germination upon induction by light. All the vegetative cells were scraped from the plate with a dull scalpel. To further eliminate the vegetative cells, the plates were exposed to chloroform vapor for 20–45 sec. The plates were then placed under low light for 24 h. The tetrads were dissected using a micromanipulator needle connected to a light microscopy under a laminar hood.

### DNA Extractions

Total DNA was extracted and purified from exponentially grown *Chlamydomonas* cells using a Qiagen Plant DNeasy Max kit (with QIAshredder). Briefly, a 200-mL culture of exponentially grown cells in TAP media was centrifuged at 600 *g* for 5 min at 4 °C. Cells were snap frozen in liquid nitrogen, pellets were briefly pulverised with a sterile mortar and pestle. The rest of the procedure was following that described by the manufacture. Extracted DNA was quantified using a spectrophotometer, and DNA purity was calculated relative to the ratio of the absorbance of A260 nm/A280 nm.

### Southern Blot

A fragment of the *AphVIII* gene was cloned by PCR (with primer AphVIII-F: (5′-CGAAGCATGGACGATGCGTT-3′ and AphVIII-R: 5′-CGAGACTGCGATCGAACGGACA-3′) and labelled with Digoxigenin-dUTP (DIG, Roche) to be used as a hybridization probe. Before hybridization, the probe was denatured by heating at 100 °C for 10 min, and then kept on ice for 5 min. Genomic DNA (~8 μg) was digested overnight by *Not*I, *Stu*I, or *Apa*I (NEB Biolabs). Digested DNA was loaded onto a 0.8% agarose gel, and each lane representing DNA cut by one specific enzyme and the gel was run slowly at 50 V to better separate fragmented DNA. Migration, transfer, and hybridization were described in detail in^52^. Detection was revealed with addition of a chemiluminescent substrate CSPD® (disodium 3-(4-methoxyspiro-3,2′-(5′-chloro)tricycle (3.3.1.13,7)decan)-4-yl)phenyl phosphate)(Roche), images were taken by G:BOX (Syngene). The number of hybridized bands in each lane indicates the potential number of *AphVIII* insertions in the genome.

### Genome Walker, Genome Re-Sequencing and Data Analyses

To identify the position of cassette insertion in the genome, genome walking PCR was carried out using a modified protocol of the commercial Genome Walker^TM^ kit (Clontech). In brief, DNA (1 μg) was digested using restriction enzymes *Fsp*I, *Nae*I, *Pml*I, *Pvu*II, or *Sma*I (New-England Labs). Digested DNA was then ligated to adapter sequences (5′-GTAATACGACTCACTATAGGGCACGCGTGGTCGACGGCCCGGGCTGGT-3′). Nested PCRs were carried out using two sets of oligonucleotides, i.e. GWaph8fw1 (5′-CTGGTGCTGCGCGAGCTGGCCCACGAGGAG-3′) or GWaph8rev1 (5′-CCAGCGCGAGATCGGAGTGCCGGTCCG-3′) with AP1 (5′-GTAATACGACTCACTATAGGGC-3′), and GWaph8fw2 (5′-TGGTTCGGGCCGGAGTGTTCCGCGGCGTT-3′) or GWaph8rev2 (5′-CGAGACTGCGATCGAACGGACACCGC-3′) with AP2 (5′-ACTATAGGGCACGCGTGGT-3′), to the cassette, and the adapter sequence in a final volume of 50 μL. PCR product was then cloned and sequenced. The obtained sequence was then put through a BLAST against the genome of *C. reinhardtii* at Phytozome^41^ to identify the flanking region.

Highly pure DNA (~15 μg per sample at a concentration of 30 ng μL^−1^; and two biological replicates were prepared; OD_260_/_280_ = 1.66) was sent to Beijing Genome Institute (BGI, China) for whole genome re-sequencing. Two Genomic read files in FASTQ (fq) format obtained from the Beijing Genome Institute (BGI, China) were uploaded to Galaxy (https://main.g2.bx.psu.edu/)^53^ and renamed “data 1” and “data 2”. Data1.fq and data2.fq were then processed using “FASTQ Groomer”, found in the NGS Toolbox Beta software package. Data were then filtered using fastQ quality filter. Processed fastq files were then joined using FASTQ joiner, also found in the NGS Toolbox Beta suite of software^40^. The *Chlamydomonas* genome v5 was uploaded as a template genome, the genome was downloaded as C_reinhardtii.110311.tar.gz from http://www.chlamy.org. Joined reads were then mapped using Bowtie with a gap length of 500 bp, and visualized with Trackster^53^,^54^.

## Additional Information

**How to cite this article**: Goold, H. D. *et al*. Whole Genome Re-Sequencing Identifies a Quantitative Trait Locus Repressing Carbon Reserve Accumulation during Optimal Growth in *Chlamydomonas reinhardtii*. *Sci. Rep.* **6**, 25209; doi: 10.1038/srep25209 (2016).

## Supplementary Material

Supplementary Information

## Figures and Tables

**Table 1:** Summary of whole genome re-sequencing results.

Insert size	Read length	Clean reads	Clean bases	Q20(%)	GC(%)
500 bp	90 bp	34257132	3083141880	92.04	60.36

Q20 is considered a high quality data commonly used by sequencing facilities. It indicates a 99% certainty that the base has been called correctly. Sequencing was performed by the Beijing Genome Institute (BGI, China).

**Table 2:**
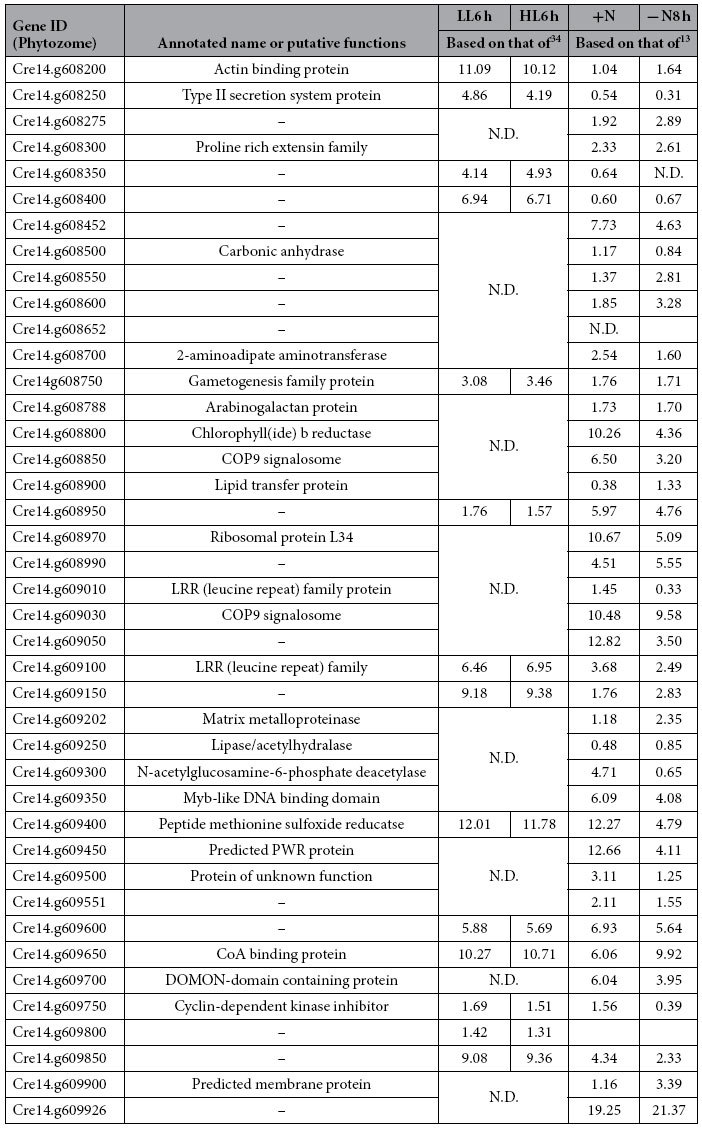
List of the putative genes encoded in the chromosome region deleted in *coa1* and their relative expression under high light or nitrogen starvation.

The listed putative genes were encoded between the coordinates 69,266 and 317,163 in chromosome 14.Values are log2-transformed average transcript reads of biological replicates for the light experiment (LL: 41 μmol photons m^−2^ s^−1^ versus HL: 145 μmol photons m^−2^ s^−1^)^34^, and are normalized mRNA abundance in RPKM (stands for: reads per kilobase of transcript per million reads mapped) for nitrogen (N) starvation experiment^13^. Abbreviations: N, nitrogen; h, hour; HL, high light; ‘−’ no annotation or no known domain identified; N.D. refers to those genes whose expression were not detected.
