# Supplementary material for: Whole Genome Re-Sequencing Identifies a Quantitative Trait Locus Repressing Carbon Reserve Accumulation during Optimal Growth in Chlamydomonas reinhardtii
Source: Sci Rep. 2016 May 4;6:25209. doi: 10.1038/srep25209 (PMC4855234; doi:10.1038/srep25209)
Supplement: Supplementary Information [file srep25209-s1.pdf]

Supplemental information for:

Whole Genome Re-Sequencing Identifies a Quantitative Trait Locus Repressing Carbon Reserve Accumulation during Optimal Growth in *Chlamydomonas reinhardtii*

Hugh Douglas Goold[1,2,3,4,#], Hoa Mai Nguyen[1,2,3,#], Fantao Kong[1,2,3], Audrey Beyly-Adriano[1,2,3], Bertrand Légeret[1,2,3], Emmanuelle Billon[1,2,3], Stéphan Cuiné[1,2,3], Fred Beisson[1,2,3], Gilles Peltier[1,2,3], Yonghua Li-Beisson[1,2,3*]

[1]CEA, BIAM, Lab Bioenerget Biotechnol Bacteries & Microalgues, Saint-Paul-lez-Durance, 13108, France

[2]CNRS, UMR 7265 Biol Veget & Microbiol Environ, Saint-Paul-lez-Durance, 13108, France

[3]Aix Marseille Université, BVME UMR7265, Marseille, 13284, France

[4]Faculty of Agriculture and the Environment, University of Sydney, Australia

Materials and methods:

Chlorophyll fluorescence: Chlorophyll fluorescence was measured using a Dual Pam-100 (Heinz Walz). Cells were placed into a cuvette at room temperature and were dark adapted for 5 min, then light curves were recorded by increasing light intensity from 0 to 600 µmol photons m^{-2} s^{-1}. Saturating flashes (10,000 µmol photons m^{-2} s^{-1}, 200 msec duration) were applied to determine the photosystem II (PSII) yield [1].

Lipid molecular species analysis by LC-MS/MS: Lipid molecular species was analyzed by LC-MS/MS following the protocol described in detail in Legeret et al [2].

Immunoblot analysis: WT and mutant cells were harvested for protein extraction and immunoblot analysis. Proteins were extracted and separated under denaturing conditions (commercial Nu-PAGE gel). Samples were loaded at equal total protein amounts based on Commassie blue staining (control). Primary antibodies (α-LHCB5 and α-AtpB) were purchased from Agrisera, and secondary anti-rabbit fluorescent antibodies (Alexa fluor) were ordered from Thermo Fischer. Control gels were run and proteins loaded were separated in the same condition and then revealed by staining with Commassie blue. LHCB5 is one of the highly conserved chlorophyll *a*/*b*-binding proteins associated with Photosystem II in algae. AtpB is the β-subunit of ATP synthase.

Supplemental Figures:

Supplemental Figure S1. Quantification of membrane lipids for the *coa1* mutant and WT.

A: Analyses of membrane lipid classes for WT and the mutant *coa1* via thin layer chromatography (TLC).

Three biological replicates with three technical replicates for each biological replicate were loaded based on an equal cellular volume basis. Lipids were run alongside purified polar lipid standards which were purchased from Larodan Fine Chemicals AB (Malmö, Sweden).

B: Comparative polar lipid analysis via liquid chromatography-tandem mass spectrometry (LC-MS/MS). Data are means of three biological replicates. Error bars denote standard deviations.

Abbreviations: TAG, Triacylglycerol; TLC, Thin Layer Chromatography; MGDG, Monogalactosyldiacylglycerol; DGDG, Digalactosyldiacylglycerol; PtdCho, phosphatidylcholine; PtdGro, Phosphatidylglycerol; PtdEtn, Phosphatidylethanolamine; PtdIns, phosphatidylinositol; DGTS, Diacylglycerol *N, N, N* –trimethylhomoserine; SQDG, Sulfoquinovosyldiacylglycerol; PtdCho: phosphatidylcholine.

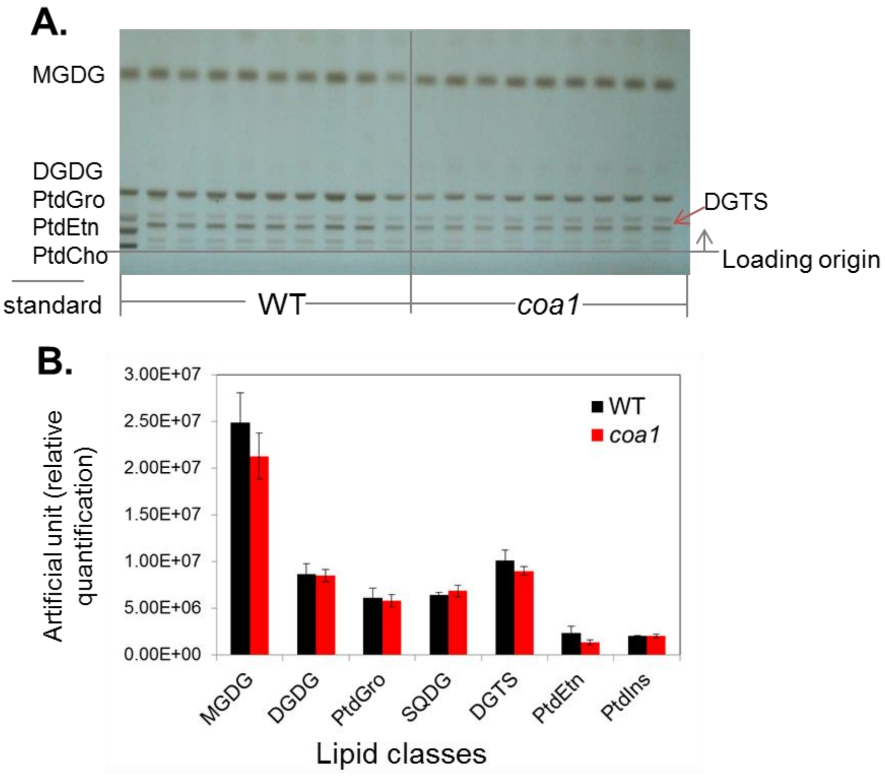

Supplemental Figure S2. Oil remobilization during the recovery phase following a period of nitrogen starvation is impaired in the mutant *coa1*.

Data are means of two biological replicates with two technical replicates each. Standard deviation is shown.

Log phase grown cells were nitrogen starved for 3 days (TAP-N), then transferred to minimal media[3] (MM+N) in the dark for up to 48 h to induce oil remobilization. Cells were harvested for oil content analyses at 0 h (TAP-N for 3 days), 24 h (MM+N) and then 48 h (MM+N).

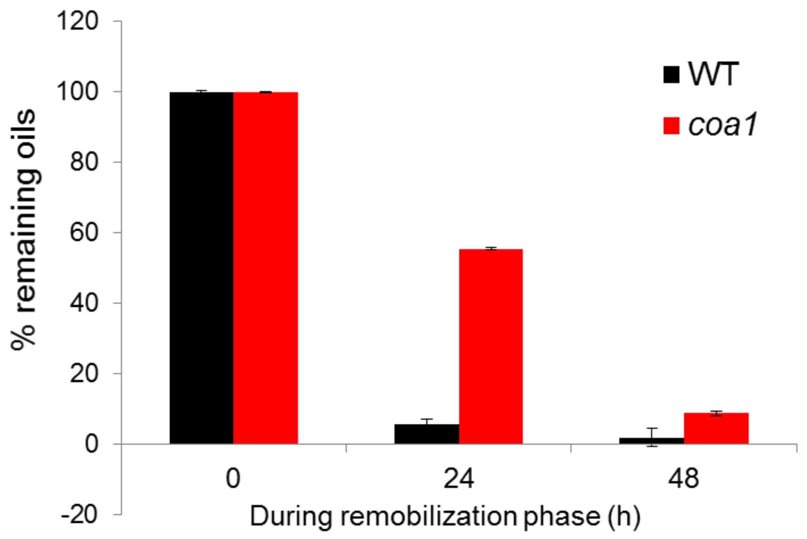

Supplemental Figure S3. Measurement of photosystem II (PSII) yield for the mutant *coa1* in comparison to WT under low light (LL) versus high light (HL) conditions.

Data are means of three biological replicates, and with standard deviations shown.

LL : 40 µmol photons $m^{-2} s^{-1}$; HL, 120 µmol photons $m^{-2} s^{-1}$

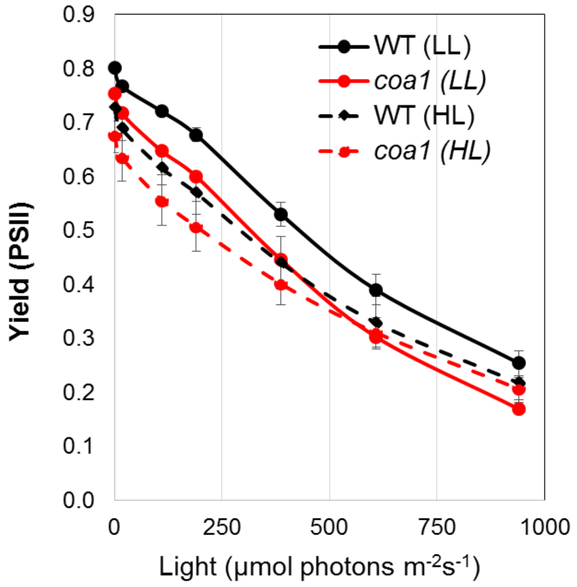

Supplemental Figure S4. The Chl *a*/*b* ratio is same between WT and the mutant *coa1* when cells were cultivated in very low light conditions (20 μmol photons m^{-2} s^{-1}).

Data are means of three biological replicates, and with standard deviations shown.

Chl : chlorophyll.

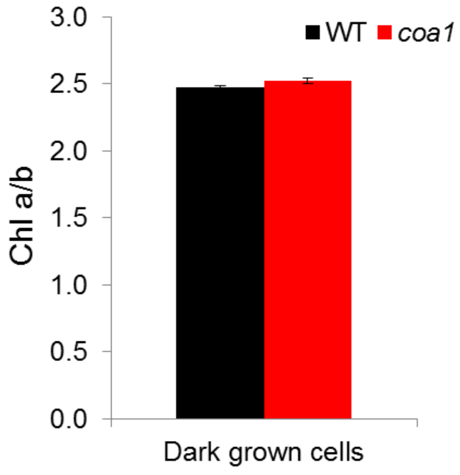

Supplemental Figure S5. Immunoblot analyses of the alterations in light harvesting complex (LHC) in the mutant *coa1* compared to WT under LL versus HL conditions.

A. Immunodetection of LHCB5 and AtpB proteins in the WT and *coa1* mutant using anti-LHCB5 and anti-AtpB antibodies, respectively.

B. Quantification of LHCB5 and AtpB proteins detected by antibodies normalized to that of WT at LL conditions (as 1).

Abbreviations: LL: low light refers to 40 μmol photons m^{-2} s^{-1}; HL: high light refers to 120 μmol photons m^{-2} s^{-1}; LHCB5: is one of the highly conserved chlorophyll *a*/*b*-binding proteins associated with Photosystem II in algae; AtpB: β-subunit of ATP synthase.

Samples (biological triplicate for each genotype) were loaded based on equal protein loading, and commercial Nu-PAGE gels were used, and proteins were separated in MES buffer, then

proteins were transferred to nitrocellulose membrane for hybridization. Primary antibodies (α-LHCB5 and α-AtpB) were purchased from Agrisera, and secondary anti-rabbit fluorescent antibodies (Alexa fluor) were ordered from Thermo Fischer. Control gels were run and separated in the same condition, but proteins were stained with Commassie blue.

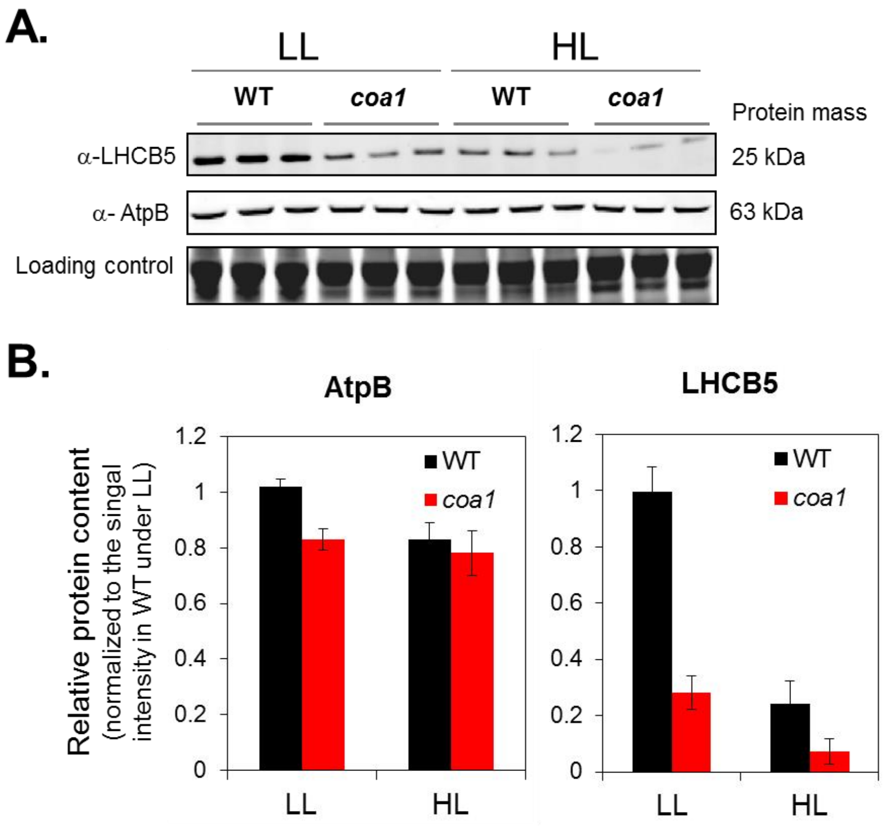

Supplemental Figure S6. Segregation analyses of the progenies of the genetic crosses between *coa1* and CC125.

A: Quantification of oil content for 9 incomplete tetrads. T: tetrad. *AphVIII* insertion-dependent of lipid phenotype is observed in the 9 incomplete tetrads analyzed.

B: Segregation phenotype of chlorophyll content in the progenies of the three complete tetrads (the same tetrads as shown in **Figure 5**). It is worth noted that T1, T2, T3 are not the same tetrads as shown in (A) above.

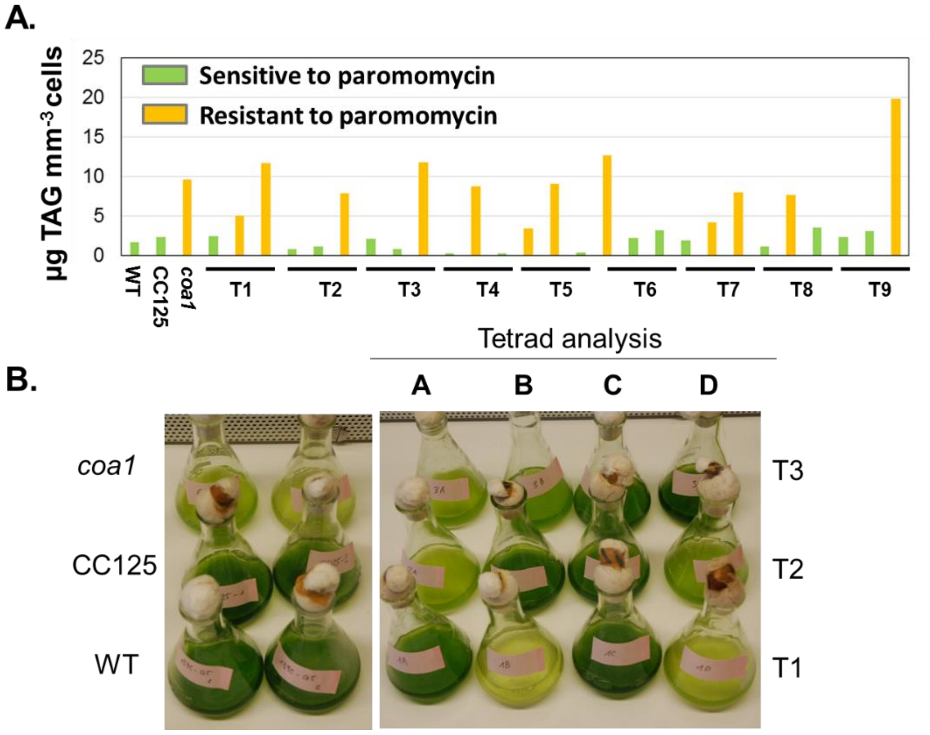

References:

1 Tolleter, D. *et al.* Control of hydrogen photoproduction by the proton gradient generated by cyclic electron flow in *Chlamydomonas reinhardtii*. *Plant Cell* **23**, 2619-2630, doi:10.1105/tpc.111.086876 (2011).

2 Légeret, B. *et al.* Lipidomic and transcriptomic analyses of *Chlamydomonas reinhardtii* under heat stress unveil a direct route for the conversion of membrane lipids into storage lipids. *Plant, Cell & Environment*, n/a-n/a, doi:10.1111/pce.12656 (2016).

3 Cagnon, C. *et al.* Development of a forward genetic screen to isolate oil mutants in the green microalga *Chlamydomonas reinhardtii*. *Biotechnology for Biofuels* **6**, 178 (2013).
